# Study of the Mechanism by Which Curcumin Cooperates with Sestrin2 to Inhibit the Growth of Pancreatic Cancer

**DOI:** 10.1155/2021/7362233

**Published:** 2021-06-30

**Authors:** Haotian Fu, Xiaofeng Ni, Fubiao Ni, Ding Li, Hongwei Sun, Hongru Kong, Yunfeng Shan, Shengjie Dai

**Affiliations:** ^1^Key Laboratory of Diagnosis and Treatment of Severe Hepato-Pancreatic Diseases of Zhejiang Province, Zhejiang Provincial Top Key Discipline in Surgery, The First Affiliated Hospital of Wenzhou Medical University, Wenzhou, Zhejiang Province, China; ^2^Department of Hepato-Pancreato-Biliary Surgery, The First Affiliated Hospital of Wenzhou Medical University, Wenzhou, Zhejiang Province, China; ^3^Department of Thyroid Surgery, The First Affiliated Hospital of Wenzhou Medical University, Wenzhou, Zhejiang Province, China; ^4^Department of General Surgery, The Second Affiliated Hospital of Wenzhou Medical University, Wenzhou, Zhejiang Province, China

## Abstract

**Background:**

Pancreatic carcinoma is a malignant tumor with a high fatality rate, and the increased resistance of pancreatic carcinoma to chemotherapy has become a difficult problem in clinical practice. Hence, it is imperative to develop an effective treatment for pancreatic cancer. Sestrins are a class of stress-induced proteins that have antioxidation functions, regulating cell growth and metabolism. Curcumin is a natural pigment isolated from turmeric. Several studies have also suggested that this molecule has multiple pharmacological effects, such as anti-inflammatory, antioxidant, and antitumor effects. However, there are insufficient studies on curcumin cooperating with the sestrin family to inhibit tumors, and the mechanism is still unclear. Our aim was to observe the potential anticancer effects of curcumin combined with the sestrin family on pancreatic carcinoma and probe its possible molecular mechanisms.

**Methods:**

Lentiviral infection, real-time fluorescence quantitative PCR assays, Cell Counting Kit-8 assays, real-time cell analysis technology, colony formation assays, wound healing assays, Transwell invasion assays, protein extraction, and western blots (WBs) were used to evaluate the effect of curcumin combined with sestrin2 on the proliferation, invasion, and migration of pancreatic carcinoma cells.

**Results:**

The results revealed that curcumin cooperated with sestrin2 to significantly suppress pancreatic cancer. In addition, we determined that sestrin2 cooperated with curcumin to inhibit pancreatic cancer by specifically targeting Nrf2/Keap1/HO-1/NQO-1.

**Conclusion:**

These findings clarify that curcumin-mediated synergistic targeting of sestrin2 is a potentially valuable treatment for pancreatic cancer.

## 1. Background

Pancreatic carcinoma is a fatal malignancy of the digestive system with a high fatality rate, accounting for roughly 432000 deaths according to GLOBOCAN 2018 estimates [1, 2]. Relevant studies have shown that pancreatic carcinoma is the seventh leading cause of cancer-related death worldwide and has the highest mortality rate among all major cancers. In the past five years, only 6% of pancreatic cancer patients have survived [3–5]. The main risk factors for pancreatic carcinoma include smoking, family history, chronic pancreatitis, diabetes, obesity, occupational exposure, a high-fat diet, Helicobacter pylori infection, etc. [6–9]. Although the causes of pancreatic carcinoma are multiplex, smoking and family history still play a dominant role. Approximately 20% of pancreatic tumors result from smoking [10].

Despite advances in current technology, surgical resection is still the only possible treatment for the disease, followed by chemotherapy [2]. Unfortunately, pancreatic carcinoma is insensitive to most chemotherapeutic agents [11]. In addition, most pancreatic carcinoma patients are diagnosed when the lesions cannot be treated with surgery [12]. Consequently, it is crucial to find an effective agent to treat pancreatic cancer with fewer side effects.

In recent years, it has been shown that sestrins are a type of stress-induced protein that is highly conserved among species, can be induced under various stress conditions, such as endoplasmic reticulum stress, and control primary cellular processes, including cell viability, antioxidant defense, cell growth, and metabolism [13, 14]. The sestrin family has three members: sestrin1, sestrin2, and sestrin3. Sestrin1, also known as the p53 activator gene, is activated in a p53-dependent manner in response to stress, such as UV and UV exposure. Sestrin2 is a vital member of the family that protects cells from a wide variety of stresses, including DNA damage, hypoxia, and oxidative stress. Sestrin3 is the third member and is activated by the forkhead box O transcription factor [15, 16]. Relevant studies have suggested that sestrin2 mainly inhibits the accumulation of reactive oxygen species (ROS) by activating the nuclear factor-erythrocyte 2-related factor (Nrf2) pathway, leading to the expression of antioxidant proteins and inhibiting the activity of mTORC1 [17]. In addition, sestrin2 plays a tumor suppressive role under normal metabolic conditions. This molecule indirectly inhibits tumor growth and activates autophagy by regulating the mTOR/AMPK signaling pathway [18, 19]. Nrf2 is an antioxidant gene inducer with good characteristics. Under normal pancreatic physiology, Nrf2 and its inhibitor, the cytoplasmic chaperone Keap1, tightly bind to inhibit its activity, while under pancreatic pathological conditions, Nrf2 and Keap1 resolve their conjugation, transfer into the nucleus, recognize and bind to antioxidant response element (ARE), and further activate heme oxygenase-1 (HO-1). HO-1 is an antioxidant gene that has a significant cytoprotective effect in pancreatic pathology. In this process, sestrin2 mainly enhances the transcription of Nrf2-related antioxidant genes by promoting the autophagic degradation of Keap1, thus achieving anticellular stress effects [20, 21].

Curcumin, a natural pigment extracted from turmeric, is the primary active ingredient of turmeric. This molecule has various pharmacological effects, such as antioxidative, anti-inflammatory, antitumor, antibacterial, free radical scavenging, and neuroprotective effects [22–24]. In addition, curcumin has been proven to be an effective Nrf2 activator that intervenes in the interaction of Nrf2-Keap1 and has a positive effect on sestrin2 in the AKT-Nrf2 pathway [25]. However, the effect of the interaction between curcumin and sestrin2 on the occurrence and development of pancreatic cancer is still unknown and should be studied for the treatment of pancreatic carcinoma mediated by traditional Chinese medicine. Since the sestrin family and curcumin have obvious tumor suppressive effects in cancer, we boldly hypothesize that curcumin and sestrin2 play an inhibitory role in pancreatic carcinoma growth.

## 2. Materials and Methods

### 2.1. Cell Culture and Reagents

The human pancreatic cancer cell lines PANC-1 and CFPAC-1 used in our study were purchased from the Cell Bank of the Chinese Academy of Sciences (Shanghai, China) and stably cultured, passaged, and cryopreserved in our laboratory. Basic Dulbecco's modified Eagle's medium (DMEM, Gibco, USA) with 10% fetal bovine serum (FBS, Sigma, St. Louis, MO, USA) was utilized to culture PANC-1 cells, while basic RPMI-1640 medium (Gibco, USA) with 10% FBS (Sigma, St. Louis, MO, USA) was utilized to culture CFPAC-1 cells, which were cultured in an incubator at 37°C, 5% CO_2_, and 95% humidity. The drug curcumin was purchased from MedChemExpress (MCE, New Jersey, USA). The anti-sestrin2 antibody was purchased from Proteintech (Chicago, USA), while the anti-Nrf2 antibody, p-Nrf2 antibody, Keap1 antibody, HO-1 antibody, and NQO-1 antibody were purchased from Abcam (Britain).

### 2.2. Lentiviral Infection and the Construction of the Cell Model

The sestrin2-overexpressing and sestrin2 knockdown lentiviruses used in this study were purchased from GeneChem (Shanghai, China). The cytotoxicity experiments were used to identify the optimal concentration of puromycin for PANC-1 and CFPAC-1 cells according to the characteristics of the puromycin resistance gene of the lentivirus. In the lentiviral transfection experiment, PANC-1 and CFPAC-1 cells were transfected with sestrin2 overexpression lentivirus and sestrin2 knockdown lentivirus, respectively, and cell models were constructed after screening with puromycin. Finally, the two cell lines were used to form a control group, a negative virus group, an overexpression group, and a knockdown group.

### 2.3. Real-Time Fluorescence Quantitative PCR Assay

PANC-1 and CFPAC-1 cells were seeded in 6 cm petri dishes (Corning, USA) at a density of 5 × 10^5^ cells/dish, and cells from each group were collected after 24 h of culture. The TRIzol reagent (Invitrogen, USA) was used to extract total cellular RNA and determine its concentration and purity. One microgram of total RNA was taken from each group for reverse transcription experiments using the Revertaid First Strand cDNA Synthesis Kit (Thermo, Manassas, USA). SYBR Green Master Mix (Biosystems, Foster, USA) was used in this q-PCR, and RT-PCR assays were carried out in a 7500 Fast real-time PCR system (USA).

### 2.4. Cell Viability Assay

PANC-1 and CFPAC-1 cell proliferation-toxicity tests and cell viability tests were performed by the CCK-8 method according to the reagent instructions. For the cell proliferation-toxicity experiment, cells were cultured in 96-well plates (5 × 10^3^/well) for 24 h and treated with puromycin at a specified concentration. Then, 10 *μ*l of CCK-8 was added after further incubation for 24 h and incubated in the incubator for 2 h. Detection of optical density (OD) at 450 nm was performed using a Multiskan spectral spectrophotometer (Thermo Fisher Scientific, USA). In the cell viability experiment, cells were inoculated in 96-well plates (5 × 10^3^/well), and the OD450 at 0 h was measured after 2-4 h of preculture. On 2-3 consecutive days, the OD450 was detected with the same CCK-8 incubation time to generate a cell activity curve.

### 2.5. Real-Time Cell Analysis

The cell culture plate E-plate 16 (ACEA, San Diego, USA) was seeded at 2 × 10^5^ cells, and the ecological index of the cells was automatically recorded on the xCELLigence RTCA TP real-time label-free cell analyzer (Agilent, California, USA). The cell index does not represent the actual number of cells. It reflects the proliferation state of cells by detecting the impedance formed by adherent cells.

### 2.6. Colony Formation Experiment

Five hundred cells/well were inoculated in a 12-well plate and cultured in an incubator. After a visible colony formed, the cells were treated with curcumin for 24 h, and then, the cells were attached with 4% paraformaldehyde and stained with crystal violet for colony counting.

### 2.7. Wound Healing Assay

The cells were seeded in 6-well plates at a density of 2 × 10^5^ cells/well and precultured for 24 h so that the cell confluence reached more than 80% of the bottom area. Then, a straight line was drawn in the center of the hole with the pipette tip vertically attached to the ruler to form a scratch. At 0 h, 24 h, and 48 h, the migration distance of the cells in the same position was observed under a microscope.

### 2.8. Invasion Assay

The invasion capacity of cells in vitro was assessed by Transwell assays. Twenty microliters of Matrigel was placed in the upper chamber of a 12-well Transwell plate (Corning, USA) and placed in an incubator at 37°C for several hours until the Matrigel became solid in the upper chamber. Cells from each group that had been prestarved were plated in Transwell plates (5∗10^4^ cells/well). In addition, 500 *μ*l of complete medium containing 10% FBS was added to the lower chamber, and serum-free basal medium (or curcumin was added to serum-free culture) was added to the upper chamber. After cultivation in a 37°C incubator for 24-48 h, the cells in the upper chamber were transferred to the lower chamber by invasion due to the serum. The upper and lower chambers were cleaned with PBS, fixed with 4% paraformaldehyde for 15 minutes, and stained with crystal violet for 10 minutes at room temperature. Finally, the cells on the inner membrane were wiped. For accurate counting, three fields were randomly selected under the microscope to calculate the number of invading cells.

### 2.9. Protein Extraction and Western Blot (WB)

After different treatments, the cells were dissolved in ice-cold RIPA lysis buffer containing 10% phosphate buffer (Basel Roche, Switzerland), 1% PMSF (Shanghai Beotem Company, China), and 1% potassium dihydrogen phosphate, and the supernatant was obtained by centrifugation for 10 min (12000 rpm, 4°C). The protein concentration was computed from the supernatant using a BCA protein detection kit (Beyotime, Shanghai, China). Before being transferred to a polyvinylidene fluoride membrane (PVDF), the total protein was subjected to 12% sodium dodecyl sulfate-polyacrylamide gel electrophoresis (SDS-PAGE). The PVDF membrane containing total proteins was sealed with 5% nonfat skim milk for 1 h at room temperature and then incubated overnight with specific antibodies at 4°C. The next day, the PVDF membrane coupled with the horseradish peroxidase- (HRP-) conjugated secondary antibody was reincubated at room temperature for 1 h after three TBST washes (7-10 min/wash). After another three washes with TBST for 5 minutes, the protein bands were detected by chemiluminescence on the autoradiography film.

### 2.10. Statistical Analysis

SPSS 18.0 (IBM, Armonk, USA) and GraphPad Prism 6.0 (GraphPad Software, Inc., San Diego, CA, USA) were used for statistical analysis (mean ± standard deviation).

Means of each pair were compared by one-way analysis of variance and the Student-Newman-Keuls test. A *P* < 0.05 was considered statistically significant.

## 3. Results

### 3.1. Construction of Pancreatic Cancer Cell Models

The optimal concentration of puromycin for PANC-1 and CFPAC-1 cells was identified via the CCK-8 method and was 1.5 *μ*M for both cell lines. After cells were infected with sestrin2 overexpression lentivirus and sestrin2 knockdown lentivirus, the cells from the negative virus group were treated with puromycin at 1.5 *μ*M, and cells from the sestrin2 overexpression group and the sestrin2 knockdown group were treated at the same concentration for 7-10 days to obtain sestrin2 overexpression and sestrin2 knockdown cell models.

### 3.2. Verification of the Sestrin2 Overexpression and Sestrin2 Knockdown Cancer Cell Groups

**Figure 1:**
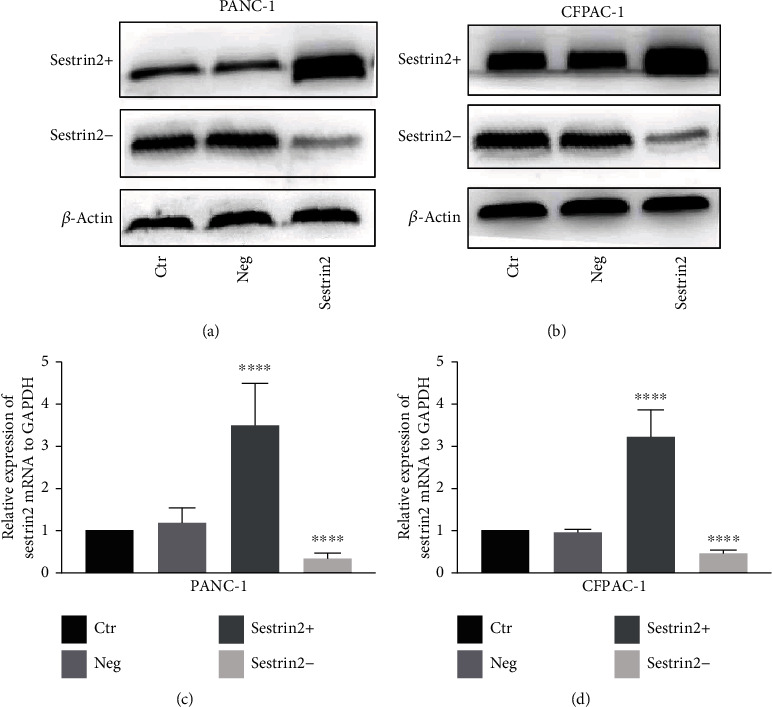
Evaluation of the pancreatic cancer cell models modified by sestrin2 overexpression/knockdown lentivirus. The expression of sestrin2 in pancreatic cancer cells infected by sestrin2 overexpression and knockdown lentivirus was visualized by western blotting (a, b), while the *β*-actin was utilized to be an internal control; the expression of sestrin2 mRNA in pancreatic cancer cells infected by sestrin2 overexpression and knockdown lentivirus was visualized by q-RT PCR (c, d).

WB and q-PCR were used to verify the expression of sestrin2 in each group. As shown in Figure 1, the expression of sestrin2 in the overexpression group was remarkably higher than that in the control group, and the expression of sestrin2 in the knockdown group was lower than that in the control group, while the expression of sestrin2 in the negative virus group was the same as that in the control group.

### 3.3. Upregulation of Sestrin2 Expression Inhibits the Invasion and Migration of Pancreatic Cancer Cells

**Figure 2:**
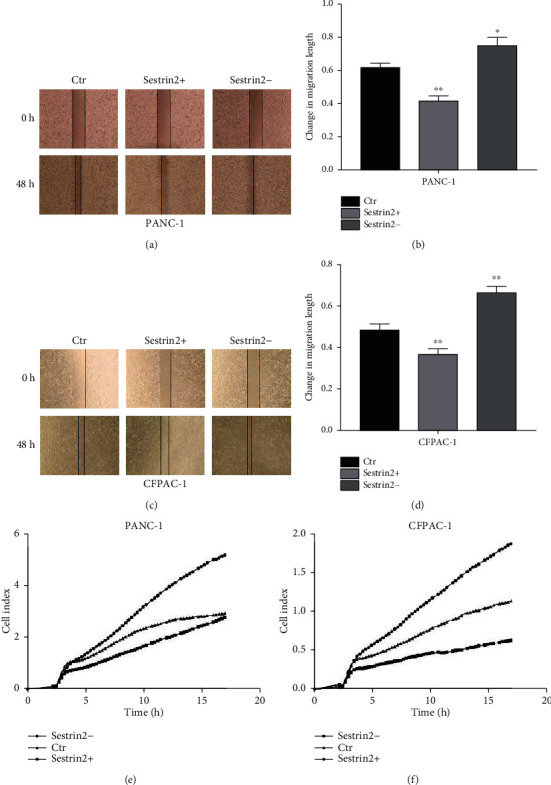
Upregulation of sestrin2 can inhibit the abilities of proliferation and migration of pancreatic cancer cells. The changes in the migration ability of the PANC-1 cell-sestrin2 overexpression group (sestrin2+) and sestrin2 knockdown group (sestrin2-) (a); the distance change of the migration is presented in the form of a histogram (b); data are represented as the mean ± SEM (*n* = 3). ^∗^*P* < 0.05 compared with control groups, ^∗∗^*P* < 0.005 compared with control groups. The changes in the migration ability of the CFPAC-1-sestrin2 overexpression group (sestrin2+) and sestrin2 knockdown group (sestrin2-) (c); the distance change of the migration is presented in the form of a histogram (d); data are represented as the mean ± SEM (*n* = 3). ^∗∗^*P* < 0.005 compared with control groups; the real-time cell analysis technology was utilized to examine the ability of proliferation of PANC-1 and CFPAC-1 cells after being infected with sestrin2 overexpression/knockdown lentivirus (e, f).

In addition, we assessed cell migration by wound healing experiments. As shown in Figures 2(a) and 2(c), the migration distance of the cells in the sestrin2 knockdown group was greater than that in the control group, while the migration distance of the cells in the sestrin2 overexpression group was lower than that in the control group (*P* < 0.05, Figures 2(b) and 2(d)). These results indicated that sestrin2 overexpression inhibited the invasion and migration of pancreatic carcinoma cells.

### 3.4. Upregulation of Sestrin2 Expression Inhibits the Proliferation of Pancreatic Cancer Cells

RTCA was used to detect the effect of sestrin2 on the proliferation of two pancreatic carcinoma cell lines. As shown in Figures 2(e) and 2(f), the proliferation curve of the sestrin2 knockdown group was faster than that of the control group, while the proliferation curve of the sestrin2 overexpression group was slower than that of the control group. These results indicated that overexpression of sestrin2 inhibited pancreatic cancer cell proliferation.

### 3.5. Curcumin Enhances the Antitumor Proliferative Effect of Sestrin2 in Pancreatic Cancer Cells

**Figure 3:**
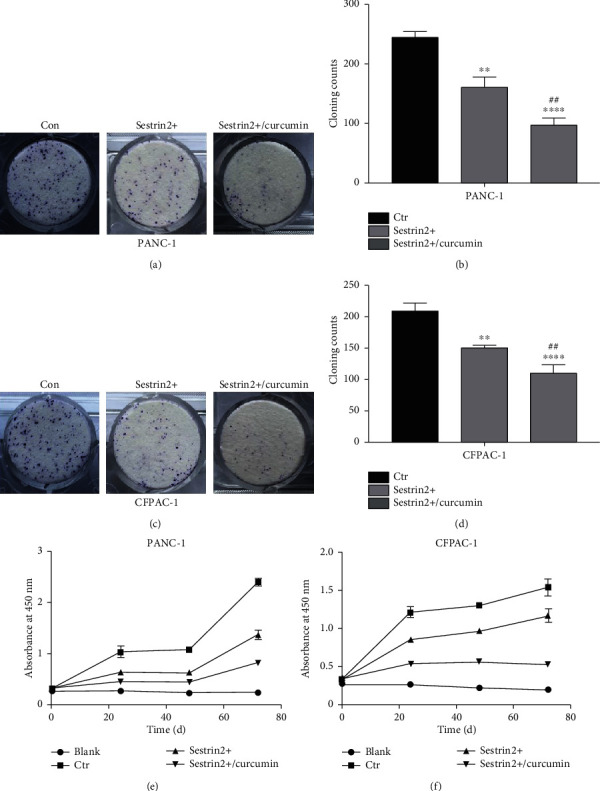
Curcumin synergistically enhances the antitumor proliferation effect of sestrin2. The changes in the proliferation ability of the PANC-1-sestrin2 overexpression group (sestrin2+) and sestrin2+/curcumin group (a); the cloning counts are presented in the form of a histogram (b). The changes in the proliferation ability of the CFPAC-1-sestrin2 overexpression group (sestrin2+) and sestrin2+/curcumin group (c); the cloning counts are presented in the form of a histogram (d); data are represented as the mean ± SEM (*n* = 3). ^∗∗^*P* < 0.005 compared with control groups, ^∗∗∗∗^*P* < 0.0001 compared with control groups, and ^##^*P* < 0.005 compared with sestrin2+ group; the CCK-8 assay was utilized to observe the change in the proliferation ability between the sestrin2+ group, sestrin2+/curcumin group, control group, and blank group in PANC-1 and CFPAC-1 cells (e, f).

The effect of curcumin on the proliferation of two pancreatic carcinoma cell lines overexpressing sestrin2 was detected by colony formation assays, which showed (Figures 3(a) and 3(c)) that the cell colony count of the sestrin2 overexpression group without curcumin treatment was less than that of the control group but larger than that of the sestrin2 overexpression group treated with curcumin (*P* < 0.005, Figures 3(b) and 3(d)). Furthermore, we determined cell proliferation by the CCK-8 method. Consistently, the proliferative ability of cells decreased significantly after treatment with curcumin (Figures 3(e) and 3(f)). These findings showed that curcumin synergistically strengthened the ability of sestrin2 to suppress the proliferation of pancreatic cancer cells.

### 3.6. Curcumin Enhances the Antitumor Migratory and Invasive Effect of Sestrin2 in Pancreatic Cancer Cells

**Figure 4:**
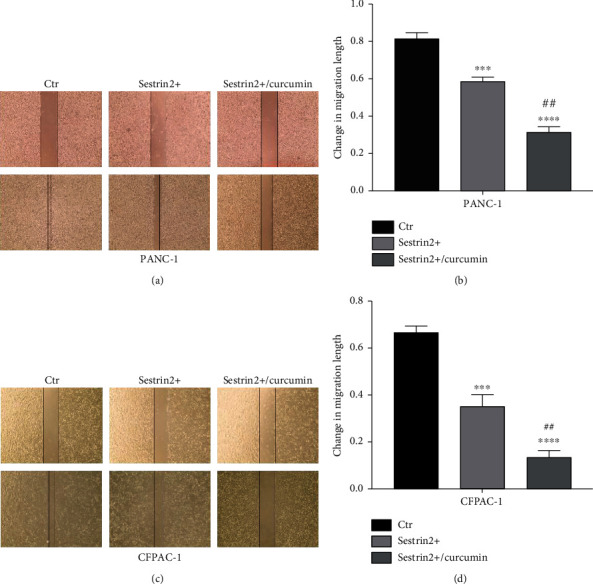
Curcumin synergistically enhances the antitumor migration effect of sestrin2. The changes in the migration ability of the PANC-1 cell-sestrin2 overexpression group (sestrin2+) and sestrin2+/curcumin group (a); the distance change of the migration is presented in the form of a histogram (b). The changes in the migration ability of the CFPAC-1 cell-sestrin2 overexpression group (sestrin2+) and sestrin2+/curcumin group (c); the distance change of the migration is presented in the form of a histogram (d); data are represented as the mean ± SEM (*n* = 3). ^∗∗∗^*P* < 0.001 compared with control groups, ^∗∗∗∗^*P* < 0.0001 compared with control groups, ^##^*P* < 0.005 compared with sestrin2+ group, and ^###^*P* < 0.001 compared with sestrin2+ group.

**Figure 5:**
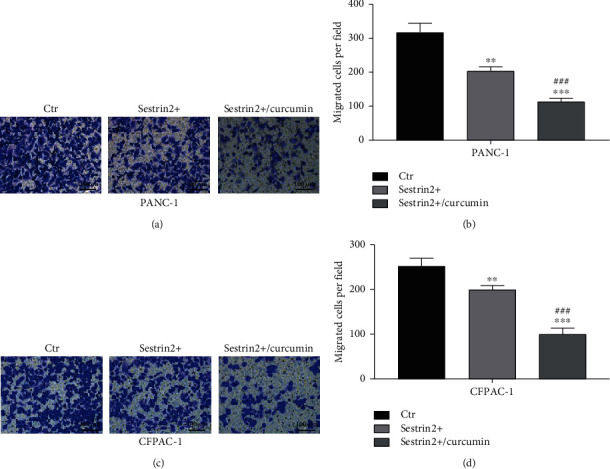
Curcumin synergistically enhances the antitumor invasion effect of sestrin2. The changes in the invasion ability of the PANC-1-sestrin2 overexpression group (sestrin2+) and sestrin2+/curcumin group (a); the migrated cell counts are presented in the form of a histogram (b). The changes in the invasion ability of the CFPAC-1-sestrin2 overexpression group (sestrin2+) and sestrin2+/curcumin group (c); the migrated cell counts are presented in the form of a histogram (d); data are represented as the mean ± SEM (*n* = 3). ^∗∗^*P* < 0.005 compared with control groups, ^∗∗∗^*P* < 0.001 compared with control groups, and ^###^*P* < 0.001 compared with sestrin2+ group.

Furthermore, we detected the effect of curcumin on the migration of the two pancreatic cancer cell lines overexpressing sestrin2 via wound healing experiments. The results are shown in Figure 4. The migration distance of the sestrin2 overexpression group without curcumin treatment was lower than that of the control group but greater than that of the sestrin2 overexpression group treated with curcumin (*P* < 0.001, Figures 4(b) and 4(d)). In addition, we assessed cell invasion by Transwell assays. Consistently, the invasive ability of cells decreased significantly after adding curcumin (*P* < 0.005, Figure 5). These results suggested that sestrin2 synergistically strengthened the ability of curcumin to inhibit the invasion and migration of pancreatic cancer cells.

### 3.7. Curcumin Enhances the Antitumor Growth Effect of Sestrin2 through the Nrf-2-Keap1/HO-1/NQO-1 Signaling Pathway in Pancreatic Cancer Cells

**Figure 6:**
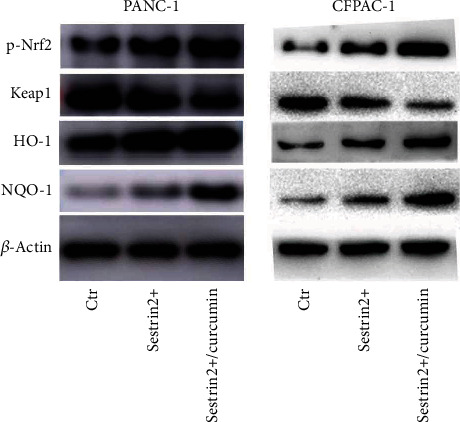
Curcumin synergistically enhances the antitumor growth effect of sestrin2 through the Nrf-2-Keap1/HO-1/NQO-1 signaling pathway. Western blotting was utilized to observe the expression of the p-Nrf2, Keap1, HO-1, and NQO-1, while the *β*-actin was utilized to be an internal control.

We further identified the possible molecular mechanism of tumor growth inhibited by sestrin2 and curcumin, and the expression of oxidative stress-related proteins in the cells with sestrin2 overexpression was determined using western blotting. As shown in Figure 6, sestrin2 overexpression increased the protein expression of p-Nrf2, HO-1, and NQO-1 in pancreatic cancer cells compared with that in the control group, and the expression of the Nrf-2 binding protein Keap1 decreased. Interestingly, we also found that curcumin treatment remarkably increased the expression of p-Nrf2, HO-1, and NQO-1 compared with that of the sestrin2 overexpression group, while Keap1 expression was remarkably decreased compared with that of the sestrin2 overexpression group. We speculated that sestrin2 cooperates with curcumin to inhibit the growth of pancreatic carcinoma through the Nrf2/Keap1/HO-1/NQO-1 signaling pathway.

## 4. Discussion

Pancreatic carcinoma is a highly malignant tumor of the digestive system, and its special tumor microenvironment is the direct cause of chemotherapy resistance. For example, gemcitabine is commonly used in clinical practice and substantially improves survival in cancer patients. However, the effectiveness of these chemotherapeutic drugs in treating pancreatic cancer is limited [26, 27]. Related studies have confirmed that low microvascular density and intense fibroinflammatory reactions are typical characteristics of pancreatic ductal adenocarcinoma (PDAC). Therefore, according to the biological characteristics of tumors, the depletion of tumor stroma seems to be a feasible treatment strategy. However, this method has contradictory results. The animal models suggested that stromal depletion with increased tumor vascularity improved the survival rate of mice, but the increase in angiogenesis was also related to tumor progression [28]. Hence, agents for treating pancreatic cancer are still restricted.

Recent studies have suggested that some natural products may be new candidates for the treatment of pancreatic carcinoma [11]. For example, the extracts of Bangladeshi medicinal plants exhibited obvious cytotoxicity against pancreatic cancer cell lines [29]. Curcumin is a compound mainly extracted from turmeric plants. The protective effect of curcumin in various diseases, including pancreatic carcinoma, has been evaluated in human studies. For example, curcumin inhibits multiple signaling pathways and suppresses cell proliferation, invasion, metastasis, and angiogenesis [30, 31]. Its wide range of medical applications includes antibacterial, analgesic, anti-inflammatory, antioxidant, antimalarial, and wound healing effects [32]. In recent years, there has been particular interest in the antioxidant and anti-inflammatory properties and suppression of angiogenesis of curcumin, which may provide a therapeutic window for the treatment of tumors [33]. In addition, curcumin has recently been used in several clinical studies, and Cheng et al. reported no treatment-related toxicity after 8 g of curcumin was taken orally daily. An experimental phase I clinical study showed that curcumin was safe even if 12 g was taken daily for 3 months [31, 34]. Recently, the curcumin analogs UBS109 and EF31 were found to downregulate the expression of angiogenic factors such as HIF-1*α*, Hsp90, and COX-2 in xenograft models of PDAC, suggesting antitumor and antiangiogenic effects [28]. In addition, curcumin restrained tumor angiogenesis in colorectal tumors [35]. Moreover, studies have confirmed that curcumin can suppress angiogenesis through the VEGF-VEGF receptor 2 signaling pathway in some types of cancer [36]. Sestrins are an evolutionarily conserved family of proteins that are primarily induced by various stressors. Of the sestrin isoforms, sestrin2 was first identified as a p53-dependent antioxidant protein that regenerated overoxidized peroxiredoxin and exhibited oxidoreductase activity in vitro, regulating cell viability, antioxidant defense, metabolism, and other major cellular processes [13]. Growing evidence indicates that sestrin2 is upregulated by oxidative stress, hypoxia, and Toll-like receptor ligands, which promote cell adaptation to stress by reducing ROS generation, inducing autophagy, and suppressing the mTOR complex [37]. However, the role of curcumin and sestrin 2 in pancreatic cancer is still unknown. In our study, we showed that sestrin2 cooperated with curcumin to inhibit the proliferation, invasion, and migration of human pancreatic cancer cells.

Alterations in signaling pathways in cells often lead to abnormal proliferation, invasion, and migration. In recent years, Nrf2, a protective antioxidant responsible for regulating cellular redox balance, has become a therapeutic target for oxidative stress. Activation of Nrf2 is a crucial strategy to control oxidative stress and inhibit the generation of ROS [38–40]. HO-1, also known as heat shock protein 32, is a cytoprotective antioxidant enzyme that can be highly induced by all kinds of compounds or different physiological and pathological conditions, including oxidant stress and hemorrhagic shock [41, 42]. Although interest in HO-1 was initially focused on its heme degradation function, relevant research results have indicated that HO-1 also has other important biological functions. Current research also shows that HO-1 plays a crucial role in the regulation of cell growth and differentiation [43]. However, the effect of HO-1 on cell proliferation is highly variable and appears to be cell type specific. For example, it has been shown that HO-1 has proproliferative effects in endothelial cells and some tumor cells, while the antiproliferative effects in vascular and airway smooth muscle cells have also been proven [44–47]. NQO-1 and HO-1 are important antioxidant enzymes in the Nrf2-ARE pathway, and NQO-1 regulates the cellular stress response by reducing quinone [48, 49]. Normally, Nrf2 is located in the cytoplasm and translocates into the nucleus after stimulation. In addition, to determine the role of sestrin2 in pancreatic cancer cells, we transfected cancer cells with the corresponding lentivirus. The results showed that sestrin2 overexpression increased the phosphorylation of Nrf2 and effectively inhibited the growth of pancreatic cancer cells. This finding was consistent with previous studies showing that sestrin2 could further activate p-Nrf2 expression by activating Keap1 autophagic degradation. Nrf2 binds to AREs in the nucleus and enhances the expression of downstream target genes, including HO-1 and NQO-1. Curcumin is a natural Nrf2 agonist that can upregulate the expression of pNrf2, negatively regulate pancreatic carcinoma cells, and suppress tumor angiogenesis. In our study, western blotting showed that curcumin-treated sestrin2-overexpressing cells had significantly increased expression levels of p-Nrf2 and further enhanced the activity of HO-1 and NQO-1, which confirmed previous research results. In summary, curcumin synergistically enhances the antipancreatic cancer growth effect of sestrin2 through the Nrf2-Keap1/HO-1/NQO-1 signaling pathway.

## 5. Conclusions

Based on several cell experiments, our results suggested that sestrin2 could effectively inhibit pancreatic cancer growth and development. Interestingly, we found that curcumin significantly enhanced the inhibitory effect of sestrin2 on pancreatic carcinoma. In addition, we further explored the potential mechanism of curcumin and sestrin2 in inhibiting pancreatic cancer and found that sestrin2 inhibits the growth of pancreatic cancer by specifically targeting the Nrf2/Keap1/HO-1/NQO-1 signaling pathway. We also found that curcumin markedly enhanced the expression of sestrin2-mediated Nrf2 and downstream target genes. These findings indicated that curcumin cooperated with sestrin2 to inhibit pancreatic cancer by specifically targeting Nrf2/Keap1/HO-1/NQO-1.

## Data Availability

The datasets used and analyzed during this study would be available upon request from the corresponding author.

## Abbreviations

WB: Western blot
CCK-8: Cell Counting Kit-8 assay
DMEM: Dulbecco's modified Eagle's medium
FBS: Fetal bovine serum
PVDF: Polyvinylidene fluoride
SDS-PAGE: Sodium dodecyl sulfate-polyacrylamide gel electrophoresis
RTCA: Real-time cell analysis technology
ROS: Reactive oxygen species
Nrf2: Nuclear factor-erythrocyte 2-related factor
HO-1: Heme oxygenase-1
mTOR: Mammalian target of rapamycin
NQO-1: NAD (P) H quinone oxidoreductase 1
ARE: Antioxidant response element
PDAC: Pancreatic ductal adenocarcinoma.
